# Clinical added value of LAFOV PET/CT in patients undergoing same-day [18 F]FDG SAFOV PET/CT scans: an initial experience report and explorative study

**DOI:** 10.1007/s00259-025-07611-6

**Published:** 2025-10-24

**Authors:** Martina Di Franco, Andrea Di Giorgio, Giulia Cuzzani, Andrea Farolfi, Paolo Castellucci, Cristina Nanni, Stefano Fanti

**Affiliations:** 1 Nuclear Medicine, Alma Mater Studiorum, University of Bologna, 40126 Bologna, Italy; 2 Nuclear Medicine, IRCCS, Azienda Ospedaliero-Universitaria di Bologna, 40138 Bologna, Italy

**Keywords:** LAFOV, PET/CT, Total-body PET, [18F]FDG, Clinical impact

## Abstract

**Purpose:**

The uEXPLORER is the first total-body, long-axial field-of-view (LAFOV) PET/CT system available for diagnostic use. The present study aims to assess its clinical impact, with a particular focus on management changes resulting from its use in routine diagnostic workflows.

**Methods:**

From June to December 2024, consecutive patients undergoing [18 F]FDG PET/CT for both oncologic and non-oncologic indications were considered for paired acquisitions using short-axial field-of-view (SAFOV) and LAFOV (uEXPLORER) systems. Scans were independently reviewed by three nuclear medicine physicians. Per-patient and per-lesion analyses were performed. Subsequent clinical management decisions were evaluated to determine the impact of LAFOV imaging. Statistical analyses included kappa statistics, Wilcoxon Signed-Rank Test, and Spearman’s correlation coefficient.

**Results:**

A total of 124 patients (64% female; median age 65.5 years) were enrolled. Inter-reader agreement was substantial for SAFOV scans (Fleiss’ k = 0.787) and almost perfect for LAFOV scans (Fleiss’ k = 0.853). In per-patient analysis, the two scans were concordant in 85% of cases (Cohen’s k = 0.73). Total lesions identified were 274 on SAFOV PET/CT and 323 on LAFOV PET/CT. Compared to SAFOV, LAFOV imaging detected 52 additional findings in 27 patients (*p* < 0.05). The per-lesion agreement was 76% (*p* < 0.05). Of the additional lesions identified by LAFOV PET/CT, 42% were confirmed as true positive. LAFOV findings led to changes in clinical management in 15% of patients, including major therapeutic changes in 68% of these cases.

**Conclusion:**

LAFOV PET/CT significantly improved lesion detection and influenced clinical decision-making. Further prospective studies are required to validate these findings and fully assess LAFOV systems clinical utility.

**Trial Registration:**

Not applicable.

## Introduction

Since its development in the 1970 s, positron emission tomography (PET) has undergone revolutionary technological advancements that have progressively expanded its role in modern medical imaging. Major innovations, such as the advent of multimodal imaging, have made the molecular insights provided by PET essential for the diagnosis and management of numerous diseases [1].

[18 F]fluorodeoxyglucose (FDG) is the most widely used radiotracer for PET combined with computed tomography (PET/CT), allowing assessment of the metabolic activity of a wide range of conditions, mostly oncologic and inflammatory. However, several well-known pitfalls and limitations can hinder lesion detectability when using [18 F]FDG in specific contexts [2, 3]. For instance, in the evaluation of slow-growing diseases, certain histological subtypes, or early-stage lesions, [18 F]FDG uptake may be low, potentially leading to false-negative results. Moreover, lesion detection can be challenging near areas of physiologically high or moderate glucose metabolism, such as the brain, the myocardium, and the liver, or in regions showing temporarily increased uptake due to functional muscular or glandular activity, as well as near the urinary tract, involved in tracer excretion [4].

The spatial resolution of short-axial field-of-view (SAFOV) digital scanners typically limits the detection of lesions smaller than 5 mm [5]. Distinguishing small lesions from artifacts can be challenging also due to signal noise that affects image quality depending on the scanner technology and the reconstruction algorithms applied [6].

The development of long-axial field-of-view (LAFOV) PET/CT systems represents one of the most significant technological advancements in molecular imaging, providing multiple advantages over SAFOV systems [7–9]. These include substantially higher count-statistics, which enables either a reduction in scan duration or a decrease in administered radiopharmaceutical activity, while maintaining excellent image quality [10].

The only total-body scanner available for clinical use is the uEXPLORER PET/CT scanner by United Imaging Healthcare (Shanghai, China), which features a 194 cm axial field-of-view and up to 40-fold higher sensitivity compared to conventional SAFOV scanners, allowing high-quality full-body imaging in a single bed-position scan for virtually all adult patients [11].

Given these capabilities, it is reasonable to hypothesize that LAFOV systems may significantly improve lesion detection and disease characterization compared to SAFOV scanners, especially in cases involving small and early-stage lesions, therefore overcoming current limitations and potentially leading to changes in patient management.

A thorough understanding of the specific clinical scenarios in which LAFOV PET systems may provide the greatest added value is essential, given the need to optimize healthcare resources when adopting novel technologies.

The aim of this study is to report our initial experience with the uEXPLORER scanner using [18 F]FDG, focusing specifically on the positivity rate compared to SAFOV systems, and to evaluate its clinical impact within a routine PET imaging center workflow.

## Materials and methods

Beginning from the implementation of the uExplorer at our institution in June 2024, until December 2024, consecutive patients scheduled for a PET/CT scan on a SAFOV scanner were considered for a subsequent acquisition using the LAFOV tomograph. Inclusion criteria were as follows: patients with oncologic or inflammatory diseases or masses of undetermined nature; age older than 18 years; availability of the LAFOV scanner immediately after the end of their first acquisition. Exclusion criteria were: patient unable to cooperate, claustrophobia, motion artifacts in the first scan, radiotracer extravasation. Patients meeting the inclusion criteria who agreed to undergo the second acquisition provided informed consent and were enrolled. A dedicated protocol regarding the use of the LAFOV scanner was approved by the local Ethics Committee (598/2024/Oss/AOUBo).

### PET/CT image acquisition protocols

All PET/CT scans were performed using [18 F] FDG in compliance with the EANM guidelines [12, 13]. All patients fasted for at least 6 h before imaging. The administered activity was 2–3 MBq/kg; uptake time of 60 ± 10 min. SAFOV PET/CT systems were GE Healthcare Discovery MI, and United Imaging Vista. The LAFOV scanner was uExplorer. Detailed acquisition protocols for each scanner utilized are provided in Table 1.

**Table 1:** Detailed PET/CT acquisition and reconstruction protocols

Scanner Type	Field of View (FOV)	Acquisition Parameters	CT Parameters	Reconstruction Parameters
GE Discovery MI (SAFOV)	25 cm	7 bed positions, 3 min/bed	120 kV, 100 mA	3D OSEM (2 iterations, 20 subsets), 6 mm Gaussian Filter, TOF, PSF
United Imaging uMI Vista (SAFOV)	24 cm	4 bed positions, 1 min/bed	120 kV, 20 mA	OSEM (2 iterations, 20 subsets) or Hyper DPR, Adaptive Filter, TOF, PSF
United Imaging uExplorer (LAFOV)	194 cm	Single bed position, 10 min	80 kV, 20 mA	OSEM (3 iterations, 20 subsets), Adaptive Filter, TOF, PSF

## Image review

All scans were anonymized and independently reviewed by three nuclear medicine physicians with at least 3 years of experience in both oncology and inflammatory/infectious diseases. In case of disagreements, a majority rule (two out of three readers) determined the final result. On a per-patient analysis, scans were categorized by each reader as positive (presence of areas of abnormal uptake, i.e. uptake visually above liver parenchyma), negative (absence of abnormal uptake) or equivocal (presence of suspicious areas of faint uptake, visually below liver parenchyma). On a per-lesion analysis, the number of the findings identified was recorded. In case of discrepancies between the two scans, the number, location, and size of discordant findings were documented.

## Follow-up

In cases where the paired scans showed discrepancies (either in overall positivity/negativity status or in the number of findings identified), the subsequent clinical decisions were recorded in order to assess whether the second scan had driven management changes or not. Management changes were categorized as major (therapy switch) or minor (additional therapeutic or diagnostic measures). When available, 6-month follow-up data were recorded and used to confirm the positivity of the findings.

### Statistical analysis

All statistical analyses were performed using R (version 4.3.1). For all statistical tests, a two-sided p-value < 0.05 was considered statistically significant.

Fleiss’ kappa was used to evaluate inter-reader agreement for each acquisition methodology. Subsequently, the concordance between the paired scans (SAFOV and LAFOV) in terms of per-patient qualitative assessment (positive, negative, or equivocal findings) was examined using Cohen’s kappa test.

To assess a potential difference in central tendency between the two methodologies, the Wilcoxon Signed-Rank Test for paired data was applied. To quantify the strength and direction of the monotonic relationship between the number of lesions detected by the two methodologies, Spearman’s rank correlation coefficient was calculated. Furthermore, the percentage of exact agreement was determined to represent the proportion of cases where both methodologies detected the exact same number of lesions. Finally, to evaluate the concordance between SAFOV and LAFOV PET/CT regarding the number of lesions, taking into account the ordinal nature of these counts and the varying severity of disagreements, weighted Cohen’s Kappa was employed, using linear weights.

## Results

A total of 124 patients were enrolled (79 females, 64%). Median age was 65,5 years, with an interquartile range (IQR) of 57–74. Median administered activity was 215 MBq (IQR = 193.7–252.3.7.3). Median uptake time was 59 min (IQR = 54–66) for SAFOV scans and 90 min (IQR = 79.5–103.5.5.5) for LAFOV scans. Patient characteristics are shown in Table 2.

**Table 2:** Patients’ population characteristics

Characteristic	(*n* = 124)
Age (years)	65.5 (IQR 57–74)
Indication for PET/CT	
Hematological malignancy	12 (10%)
Lung cancer	3 (2%)
Breast cancer	18 (15%)
Gynecological cancer	18 (15%)
Pancreatic cancer	8 (6%)
Pulmonary nodules	11 (9%)
Inflammation	14 (11%)
Other pathologies	40 (32%)
SAFOV Acquisition Details	
GE MI	83 (67%)
United Vista	41 (33%)
Uptake Time (min)	
SAFOV scans	59 (IQR 54–66)
LAFOV scans	90 (IQR 79.5–103.5)
Administered Dose (MBq)	215 (IQR 193.7–252.3)

Continous data are median and interquartile range

The inter-reader agreement was substantial for SAFOV scans (Fleiss’ k = 0.787), and almost perfect for LAFOV scans (Fleiss’ k = 0.853); unanimity was reached for 103(83%) SAFOV and 110(88%) LAFOV scans.

**Fig. 1:**
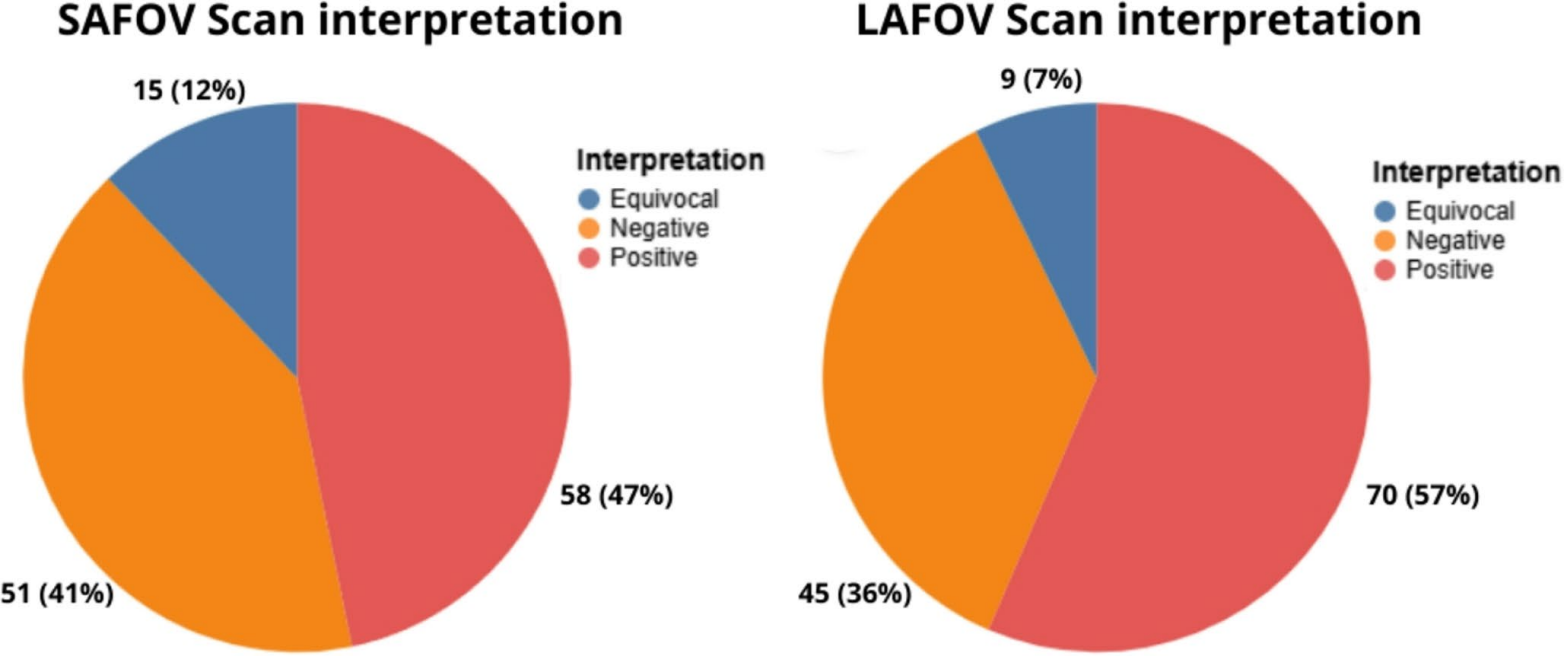
The pie charts illustrate the percentage distribution of scan interpretations (Negative, Positive, Equivocal) for both SAFOV and LAFOV acquisitions

In a per-patient analysis, the SAFOV PET was positive in 58(47%) patients, negative in 51(41%) and equivocal in 15(12%); the LAFOV PET was positive in 70(57%) patients, negative in 45(36%), and equivocal in 9(7%)(Fig. 1). The two paired acquisitions were concordant in 105(85%) cases: they were both positive in 57(54%), both negative in 42(40%) and both equivocal in 6(6%). The paired scans were discordant in 19 cases: 6(32%) were SAFOV-negative and LAFOV-positive, 3(16%) were SAFOV-negative and LAFOV-equivocal, 7(37%) were SAFOV-equivocal and LAFOV-positive, 2(10%) were SAFOV-equivocal and LAFOV-negative, 1(5%) was SAFOV-positive and LAFOV-negative. No cases of positive SAFOV and equivocal LAFOV PET/CT were recorded. The per-patient concordance was 84,7%, with a Cohen’s kappa = 0.73, indicating substantial agreement.

**Fig. 2:**
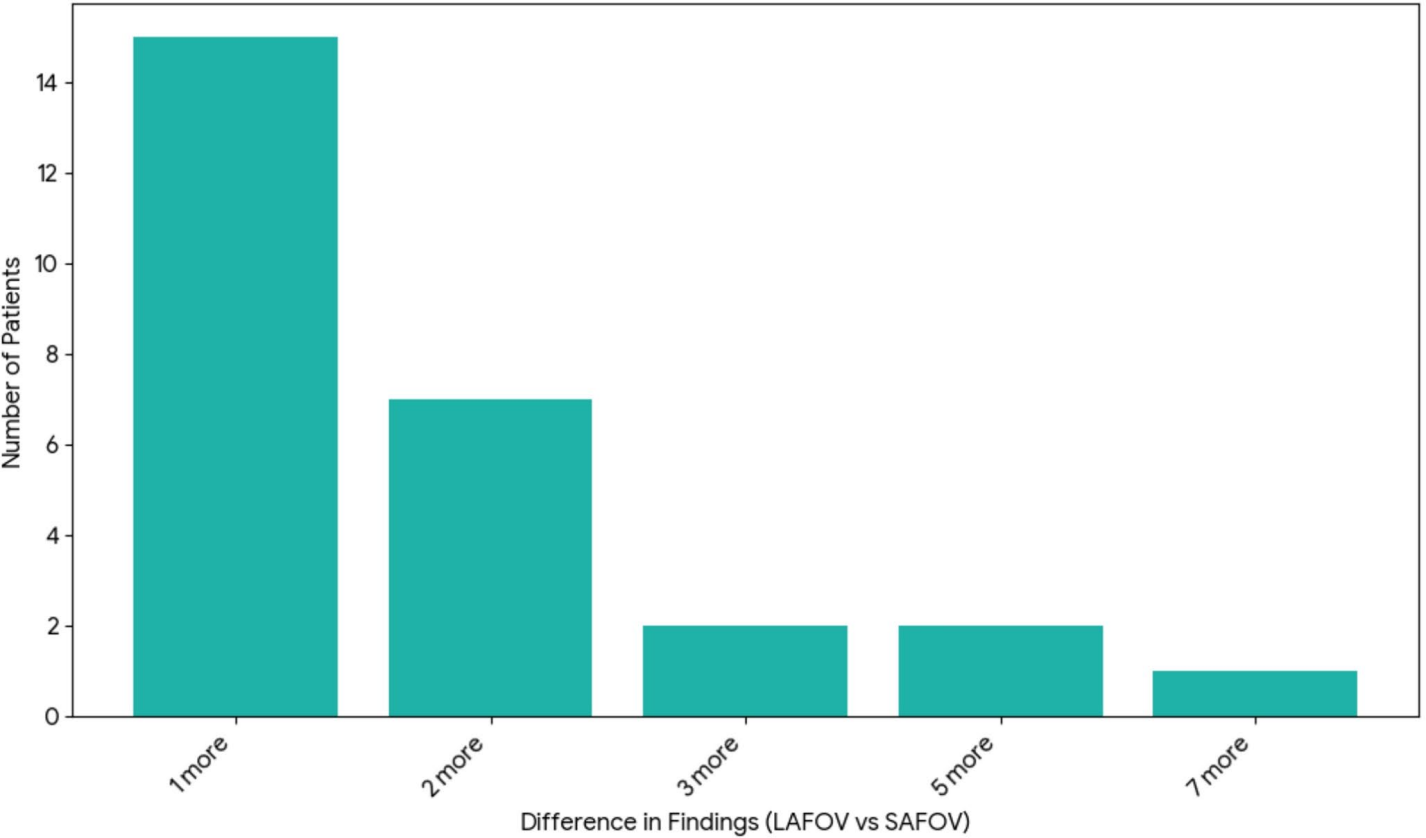
Number of Patients by Additional Lesions Detected by LAFOV

A total of 274 lesions were identified on the SAFOV and 323 on the LAFOV scans. In 94/124(76%) patients, the two acquisitions showed the exact same number and sites of findings. In the remaining 30(24%), the two scans showed a different number of findings: compared to the SAFOV, LAFOV PET/CT identified a total of 52 additional findings in 27 patients. Specifically, LAFOV PET/CT detected 1 more finding in 15 patients (*n* = 15 findings), 2 more in 7 patients (*n* = 14 findings), 3 more in 2 patients (*n* = 6 findings), 5 more in 2 patients (*n* = 10 findings), 7 more in 1 patient (*n* = 7 findings). In 3 patients, the SAFOV identified one more finding compared to the LAFOV PET/CT (Fig. 2) (Table 3).

**Table 3:** Imaging Findings, Concordance, and management changes by pathological category

Pathological Category	No. of Patients	SAFOV Findings	LAFOV Findings	Concordance (No.)	Concordance (%)	Change in Management (No., %)
Hematological malignancy	12	26	30	9/12	75%	0
Lung cancer	3	20	20	3/3	100%	0
Breast cancer	18	48	47	17/18	94%	0
Gynecological cancer	18	19	31	12/18	67%	5 (4%)
Pancreatic cancer	8	27	32	5/8	63%	3 (2%)
Pulmonary nodules	11	21	24	8/11	73%	3 (2%)
Inflammation	14	18	20	13/14	93%	0
Other pathologies	40	95	119	27/40	68%	8 (7%)
Total	124	274	323	94/124	76%	19 (15%)

Of the 27 patients in which the LAVOF PET/CT identified additional findings, 15(56%) were SAFOV positive, 3(11%) were SAFOV equivocal, 9(33%)% were SAFOV negative.

The Wilcoxon Signed-Rank Test p-value for the difference in number of lesions between SAFOV and LAFOV PET/CT was < 0.05. The Spearman’s rank correlation coefficient for the number of lesions was 0.891, with a p-value < 0.05. The weighted Cohen’s Kappa (linear weights) for the number of lesions was 0.927. The percentage of agreement for the number of lesions was 76%.

**Fig. 3:**
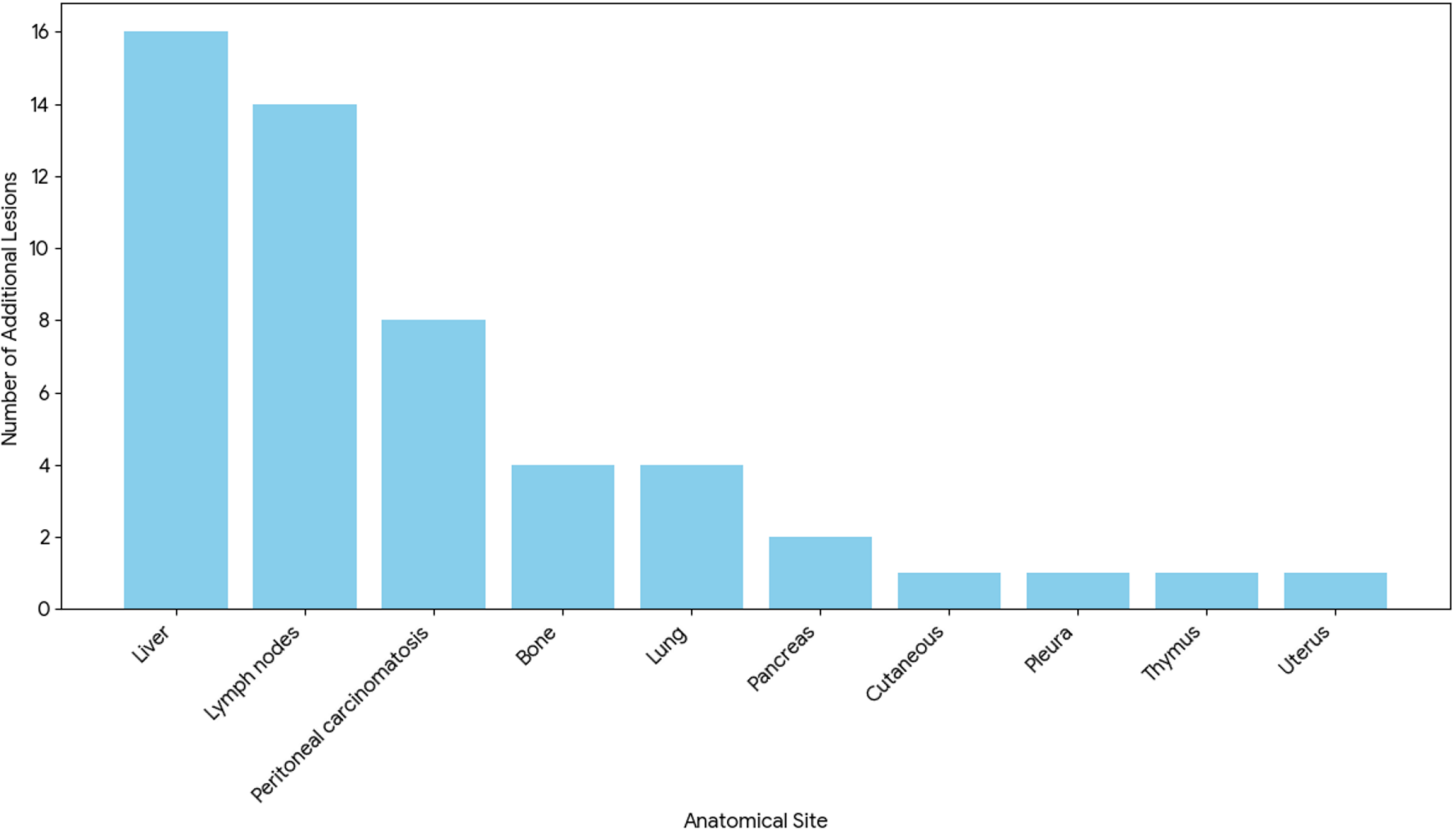
Number of Additional Lesions Detected by LAFOV per Anatomical Region

The sites of the additional findings detected on the LAFOV scan are shown in Fig. 3. When measurable on corresponding low-dose CT, all additional findings measured less than 1 cm in their major diameter.

**Fig. 4:**
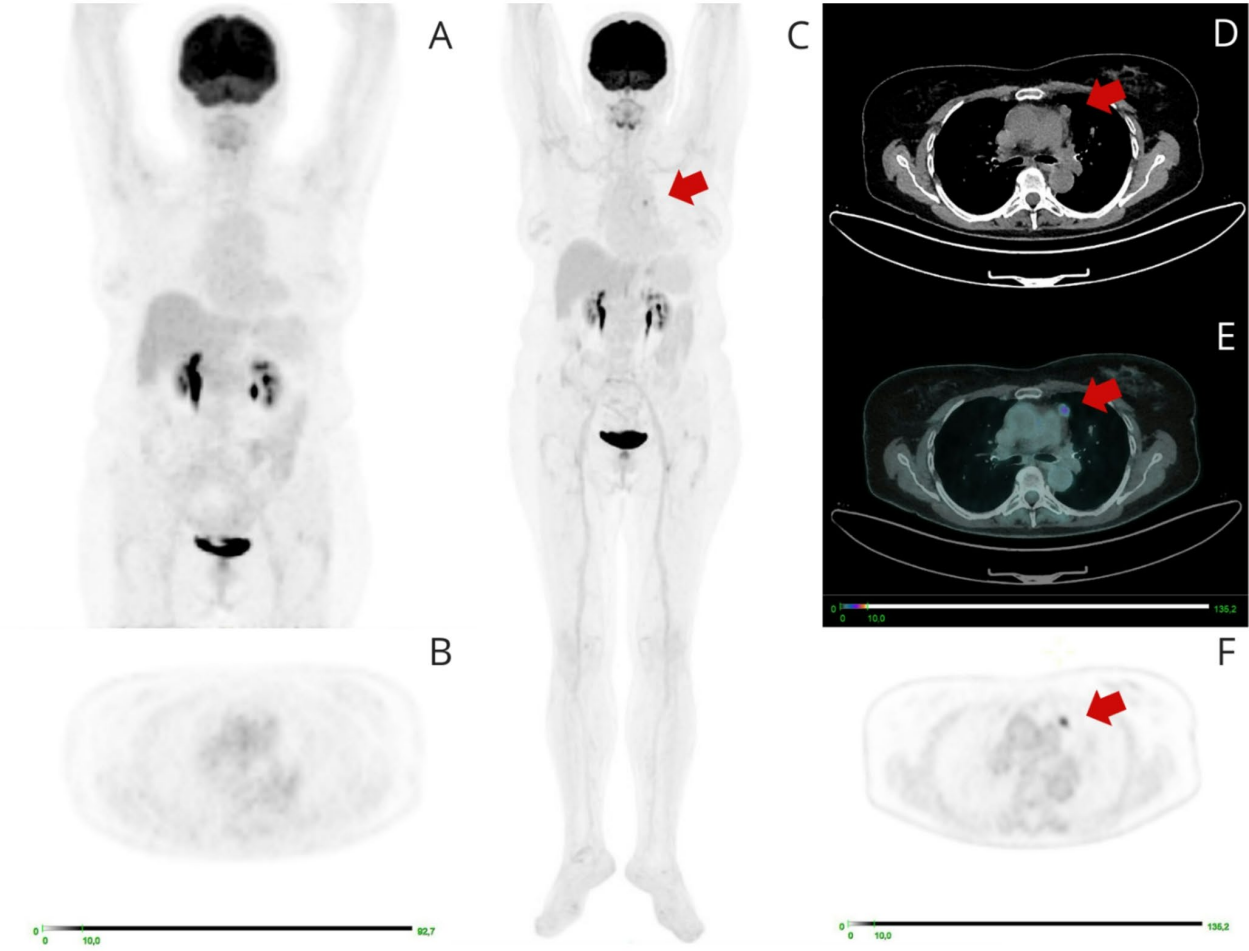
[18 F]FDG PET/CT performed for restaging in a patient with a history of ovarian carcinoma. The patient underwent bilateral hystero-adnexectomy and adjuvant chemotherapy. Ten years later, an increase in CA 125 levels to 45 ng/mL (normal value < 35 ng/mL) was noted. The SAFOV PET/CT scan (**A**) demonstrated increased FDG uptake in the VI hepatic segment (**G**). Subsequent LAFOV PET/CT scan (**B**) confirmed the presence of the lesion in the VI hepatic segment (**H**) and revealed two additional hepatic uptake areas: one at the dome of the VIII segment (**D**), another in the VI segment (**F**). Additionally, uptake was noted in a lymph node of the right internal mammary chain (**D**). The patient subsequently underwent surgical removal of the hepatic nodules, which were confirmed to be secondary metastatic localizations

Of the 52 findings identified at the LAFOV but not on the SAFOV PET/CT, 22(42%) were true positive (*n* = 7 based on subsequent histopathological analysis, *n* = 15 based on other imaging modalities) (Fig. 4), while 30(58%) were not evaluable due to the lack of a confirmation method. The 3 lesions identified by the SAFOV but not detected on the LAFOV scan were considered false positives on the basis of the follow up (non-detectable at subsequent PET/CT scans).

**Fig. 5:**
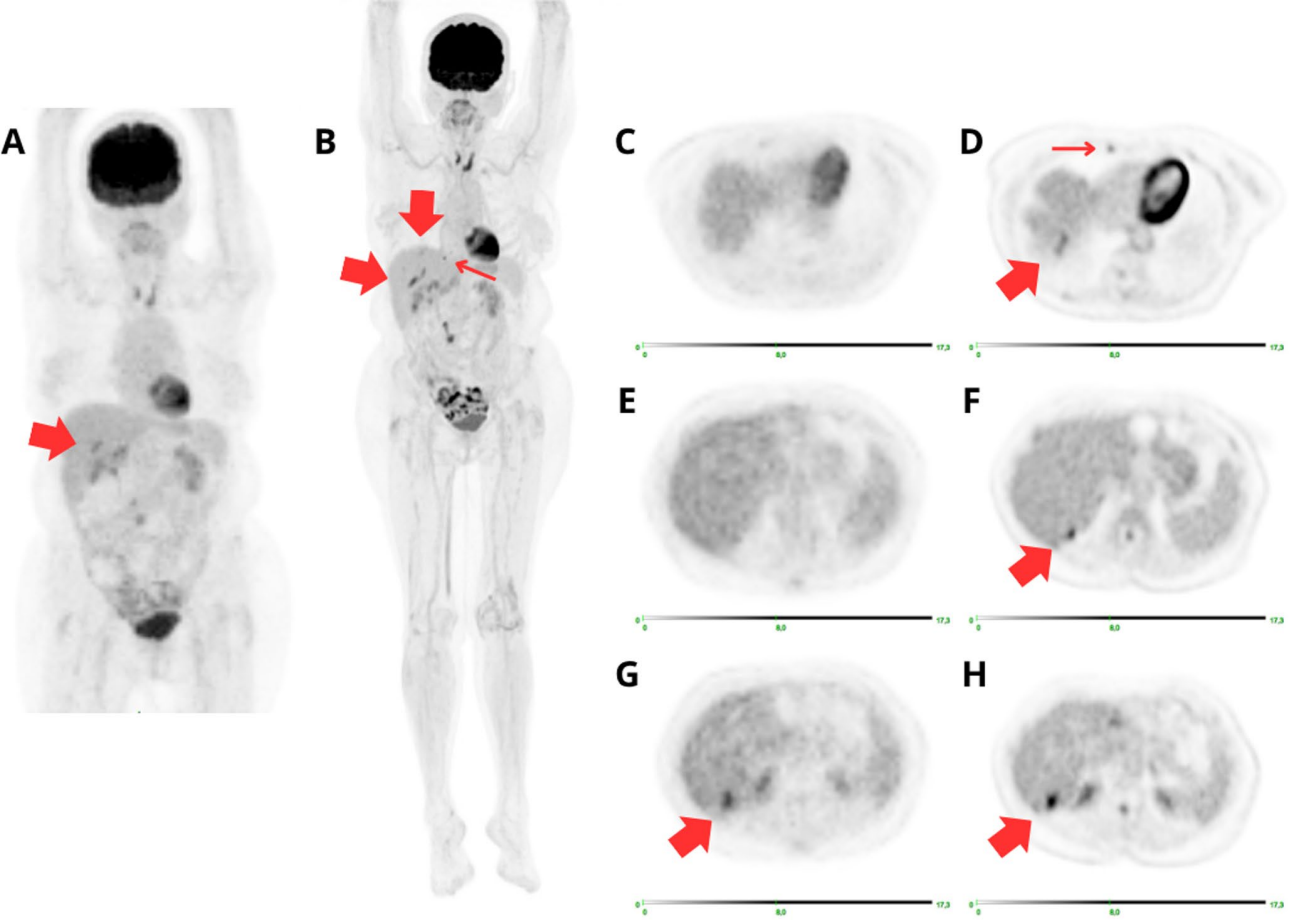
A 73-year-old woman with a CT finding of a para-mediastinal nodule. The [18 F]FDG PET/CT SAFOV scan (**A**) is negative, while the total-body scan (**B**) shows focal uptake corresponding to the nodule. Surgical removal of the nodule established a diagnosis of thymic carcinoma

The additional information obtained from the LAFOV acquisition led to changes in the therapeutic management of 19(15%) patients (Fig. 5). Major changes were reported for 13/19(68%) patients and minor changes for 6/19(32%). Detailed changes of management are reported in Table 4.

**Table 4:** Management changes

SAFOV – LAFOV						
Case n°	**Condition**	**PET Indication**	**SAFOV Findings**	**LAFOV Findings**	**Change of Management**	**Confirmation Method**
1	Pulmonary Nodule	Metabolic Characterization	None	Positive pulmonary nodule	Minor: biopsy	Histological diagnosis of adenocarcinoma
2	Pulmonary nodule	Metabolic characterization	Negative	Positive pulmonary nodule	Minor: biopsy	Histological diagnosis of adenocarcinoma
3	Pulmonary nodule	Metabolic characterization	Negative	Positive pulmonary nodule	Minor: biopsy	Unsuccesful biopsy, negative BAL
4	Incidental finding of pleural thickeining and peritoneal nodulations	Metabolic characterization	None	Positive pleural thickening	Major: Start of first line of chemotherapy	Follow up. No evidence of disease progression.
5	Incidental finding of a para-mediastinal node	Metabolic characterization	None	Positive para-mediastinal node (Fig. 5)	Major: Surgery	Histological diagnosis of thymic carcinoma.
6	Pharyngeal cancer	Staging	Focal uptake al primary site	Primary tumor and laterocervical nodes	Minor: RT boost on lymph nodes	Follow up. No evidence of disease progression
7	Colon carcinoma	Suspected liver lesions	Liver lesions	Positive liver lesions and Celiac lymph node	Major: Wedge resection and lympadenectomy	Histological diagnosis of colon carcinoma metastases
8	Colon carcinoma	Suspected liver lesions	Negative	Positive Liver lesion	Major: Radiofrequency	Finding positive on CEUS
9	Colon carcinoma	Staging	Uptake on the primary tumor	Positive Liver Lesion	Major: Start of first line of chemotherapy	Positive liver finding on CT and MRI
10	Colon carcinoma	Staging	Positive on single liver lesion	Positive on more liver lesions	Major: Start of first line of chemotherapy instead of local treatment	Confirmed positive liver findings on CT
11	Colon carcinoma	Suspected recurrence	Negative	Positive liver lesion	Major: first line of chemotherapy	Confirmed positive liver finding on CEUS and progression on subsequent PET/CT
12	Pancreatic Cancer	Suspected recurrence	None	pancreatic focalities	Major: first line chemotherapy	Confirmed positive pancreatic findings on endoscopic ultrasound
13	Pancreatic Cancer	Suspected recurrence	None	2 pancreatic focalities	Major: Start of first line chemotherapy	Confirmed positive pancreatic findings on endoscopic ultrasound
14	Pancreas	Suspected recurrence	Positive on liver lesion	Negative	Major: FU instead of surgery or RF	Negative follow-up imaging (MRI)
15	Ovarian Cancer	Staging	Uptake of the primary tumor and peritoneal nodules	Same as SAFOV, in addition: uptake of a pulmonary nodule	Major: SBRT	Confirmed positive peritoneal findings con CT
16	Ovarian Cancer	Elevated tumor markers during FU	None	Positive Liver Lesion (Fig. 4)	Minor: biopsy	Histological diagnosis of ovarian cancer metastasis
17	Ovarian Cancer	FU	Negative	Positive on left supraclavicular lymph node (Fig. 6)	Minor: biopsy	Histological diagnosis of ovarian cancer metastasis
18	Ovarian Cancer	Suspected recurrence	Positive on liver lesion	Negative	Major: FU instead of surgery or RF	Negative follow-up imaging (PET/CT)
19	Ovarian Cancer	Suspected recurrence	Positive liver lesion	Negative	Major: FU instead of surgery or RF	Negative follow-up imaging (PET/CT)

## Discussion

In recent years, significant efforts have been made to enhance the accuracy and image quality of PET/CT imaging, including the transition from analog to digital systems and the development of advanced image reconstruction algorithms [14–16]. Nonetheless, diagnostic challenges persist. A key issue in PET/CT is the accurate metabolic or molecular characterization of small findings or lesions at early stage [17]. The sensitivity of current SAFOV PET scanners is inherently limited by the 20–30 cm field-of-view (FOV), meaning that 80–90% of the body remains outside the FOV during the scanning time, preventing the detection of several decay events. By extending the FOV and implementing latest generation detectors, LAVOF systems provide enhanced collection efficiency, resulting in a higher signal-to-noise ratio, the possibility of reducing the administered activity and/or scan time [18, 19]. By the same principle, when the full activity is administered, an improved lesion detection can be hypothesized.

The key result in our series is the higher number of additional findings detected by the LAFOV scanner in approximately one fifth of the population. Specifically, the Wilcoxon Signed-Rank test demonstrated a statistically significant difference between the two modalities considering the distribution of additional findings; paired scans frequently identified a different number of findings, with a tendency in favour of the LAFOV system. Nevertheless, in the per-lesion analysis, the weighted Cohen’s Kappa value revealed almost perfect agreement between the two scanners, meaning that, considering the “extent” of the disagreements, the overall concordance was very high. In other terms, the difference was attributed to relatively small systematic differences, rather than large discrepancies, with the LAFOV system often detecting one or few more findings than the SAFOV one. The very high Spearman’s rank correlation coefficient further supports a strong relationship in lesion detection.

**Fig. 6:**
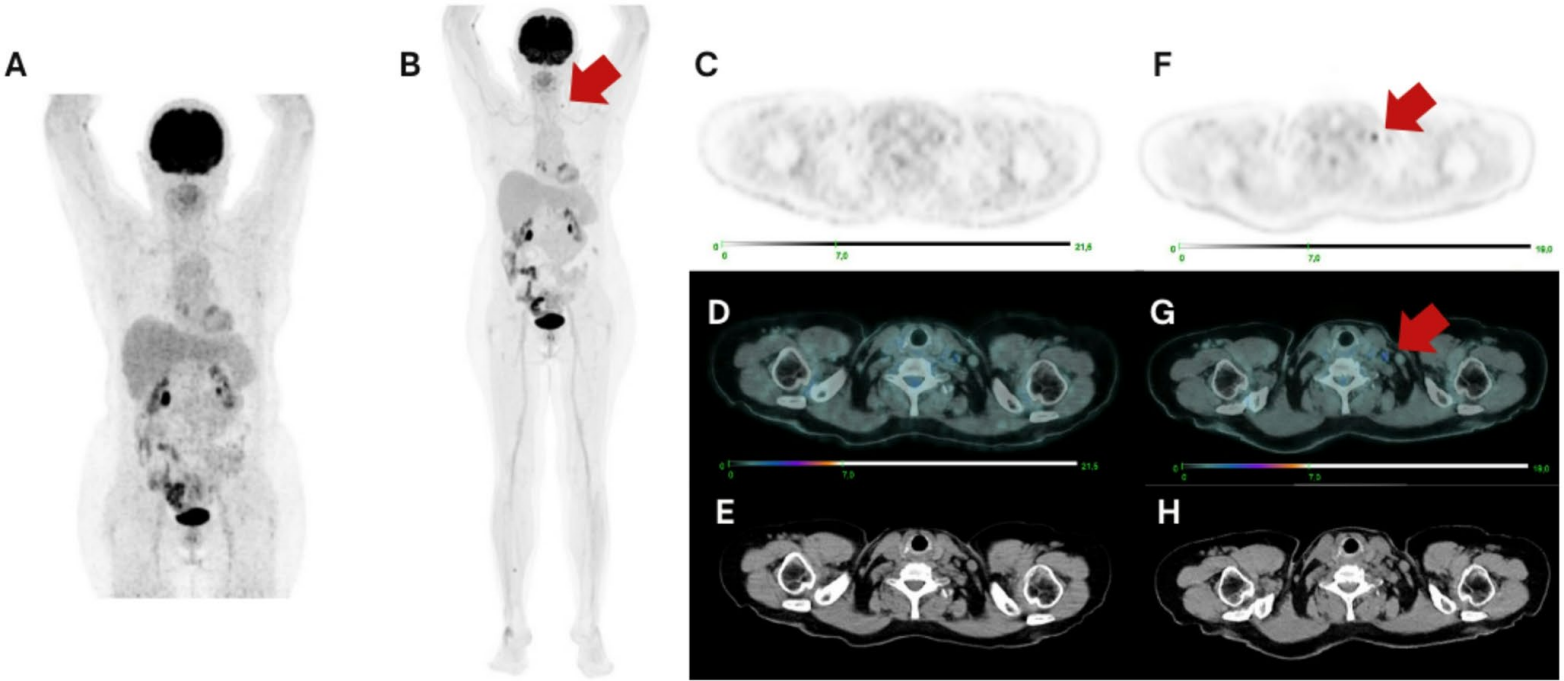
[18 F]FDG PET/CT performed for restaging in a patient with advanced high-grade ovarian cancer, with previous peritoneal localizations, currently undergoing treatment with niraparib. The SAFOV examination is negative (**A**, **C**-**E**), while the LAFOV PET/CT reveals low level uptake in a small left supraclavicular lymph node (**B**, **F**-**H**). Biopsy of this lymph node confirmed metastatic ovarian carcinoma

When a confirmation method was available, all the additional lesions detected on the LAFOV PET/CT were true positive (42% of the total) (Fig. 6). These results are relevant since even small numeric discrepancies in lesion detection might translate into major clinical benefits [20].

Chen et al. conducted a head-to-head intra-individual comparison between the uEXPLORER and a digital SAFOV PET/CT in 67 oncological patients undergoing a single [18 F]FDG administration, focusing on lesion detectability and image quality. Their study demonstrated significantly higher subjective image quality and signal-to-noise ratio (SNR) for the uEXPLORER, which identified 15 additional lesions compared to the SAFOV system (*n* = 241 total lesions) [18]. However, the clinical implications of this superior lesion detection were not addressed.

The impact of LAFOV PET/CT in lung cancer staging has been addressed in a recent prospective study by Mingels et al., who reported high diagnostic accuracy for both LAFOV and SAFOV systems, without statistically significant differences, and non-significant slightly higher sensitivity and positive predictive value on the LAFOV compared to SAFOV PET/CT [21]. Similarly, in a retrospective study by Alberts et al., comparable diagnostic accuracy was observed between SAFOV and LAFOV systems in the assessment of non-small cell lung cancer [22].

These results support the assumption that LAFOV PET/CT may offer less additional value in patients with high-grade neoplasms, as high metabolic lesions are already well characterized by SAFOV systems. The proportion of patients who might benefit from the detection of one or few additional lesions has been estimated at 4.5% by Dragonetti et al., based on a single-center analysis of clinical records. The study identified patients presenting with 1 to 2 FDG-positive lesions and an SUVmax of the most avid lesion between 2 and 5. Patients with these characteristics were considered likely to benefit from the detection of even a slightly different lesion count [23].

In the population analyzed in this study, the increased sensitivity of LAFOV PET/CT led to a change in management in 15% of patients. This included both major clinical decisions—such as surgery or therapy modification—and minor interventions like biopsies or radiotherapy boosts.

As expected, all the additional findings detected were small, sub-centimetric when measurable.

The most notable impact was observed in the evaluation of the liver, either through the identification of additional lesions or the resolution of small foci. Specifically, among the 19 patients whose management was altered based on the second LAFOV acquisition, 6 had additional hepatic lesions, while in 3 cases the liver parenchyma appeared normal after the LAFOV scan, and the corresponding 3 findings detected on the SAFOV were recognized as false positives through subsequent imaging.

Assessing the liver can be particularly challenging due to the physiologic background signal, which is often heterogeneous and may either mimic pathological uptake or obscure true lesions when using various radiopharmaceuticals [24, 25]. For this reason, and in light of these preliminary results, this specific setting warrants further investigation.

Currently, studies evaluating the impact of LAFOV PET/CT on patient management using [18 F]FDG are still limited. On the other hand, the areas of investigation for PSMA PET/CT appear more straightforward, particularly given the clinical need to improve the detection of biochemical recurrence (BCR) at lower PSA levels after prostatectomy. Indeed, some studies have already demonstrated a patient benefit from LAFOV imaging in this setting [26, 27].

The 15% rate of management change, a direct result of the enhanced lesion detection, is a highly encouraging finding, also in terms of cost-benefit, as highlighted by the recent analysis of Alberts et al. [28], which demonstrated the superior cost-effectiveness of LAFOV systems. It reflects the technology’s capacity to provide a more comprehensive, “total-body” assessment with unprecedented sensitivity, leading to more accurate clinical decisions and potentially improved patient outcomes.

Given the current unmet need to define the most appropriate clinical applications of LAFOV system for [18 F]FDG PET/CT, we believe that this exploratory, hypothesis-generating study may stimulate further research, despite its inherent limitations.

The main limitation is that the LAFOV acquisition was always performed after the scan on the SAFOV system. This sequence was necessary during the implementation phase of the new scanner. Moreover, the lack of randomization exposes the study to selection biases. Randomization was not feasible due to ethical and practical constraints: it would have been inappropriate or impossible to randomly assign patients to the second acquisition respecting the availability of the scanners, patient preferences with regard to the new system, or their ability to cooperate.

Delayed acquisitions are part of routine clinical practice in many PET centers and have often been helpful in improving the interpretation of nonspecific findings [29]. However, differences of this magnitude in lesion count have rarely been reported in the literature since the advent of new digital tomographs [30, 31]. We therefore consider this finding to be noteworthy, despite the limitations described above. Nevertheless, due to the different uptake times, we decided not to include semiquantitative parameters in our analysis.

Additional challenge was the logistical constraints inherent in a high-volume PET center, which limited our ability to enroll a larger cohort of patients. The availability of the LAFOV scanner immediately after a patient’s initial SAFOV scan was not always guaranteed, as the tomograph was often in use for other scheduled appointments. While most patients were willing to participate, a small number declined for personal reasons, such as travel distance or time constraints. Other limitations include the small patient population per clinical entity and the lack of validation for all the positive findings, especially for small lesions that are not feasible to biopsy. Long-term follow up and extended cohorts would allow to overcome these limits.

Our preliminary results on LAFOV PET clinical impact raise ethical considerations that will be worthy of future evaluation. A crucial ethical issue is which scanner to assign to each patient in a department with both SAFOV and LAFOV systems. As clear guidelines for the optimal clinical applications of LAFOV are currently lacking, we believe this ethical dilemma underscores the critical need for further research. Our study was designed to provide initial, hypothesis-generating data by directly comparing the two modalities in a real-world clinical setting. We hope our findings, combined with future studies on large populations, will help identify the specific clinical scenario where LAFOV system offers a clear diagnostic advantage. This, in turn, will provide the necessary evidence to develop ethical and clinically informed protocols for patients’ assignment, ensuring each patient receives the most appropriate and beneficial imaging modality.

## Conclusion

Our preliminary single-institution study suggests that LAFOV PET/CT consistently outperformed SAFOV PET/CT in lesion detectability in late acquisitions, prompting therapeutic changes; however, the potential influence of the later acquisition time on these results should be considered. These findings, while promising, warrants further inverstigation in prospective studies with larger patient populations and long-term follow-up are needed to validate the results and define the optimal clinical applications of LAFOV PET imaging.

## Data Availability

The datasets generated during and/or analyzed during the current study are available from the corresponding author for reasonable requests. This study was performed in line with the principles of the declaration of Helsinki.
